# Therapeutic efficacy trial of artemisinin-based combination therapy for the treatment of uncomplicated malaria and investigation of mutations in *k13* propeller domain in Togo, 2012–2013

**DOI:** 10.1186/s12936-016-1381-8

**Published:** 2016-06-22

**Authors:** Améyo M. Dorkenoo, Degninou Yehadji, Yao M. Agbo, Yao Layibo, Foli Agbeko, Poukpessi Adjeloh, Kossi Yakpa, Efoe Sossou, Fantchè Awokou, Pascal Ringwald

**Affiliations:** Faculté des Sciences de la Sante, Université de Lomé, BP 1515 Lomé, Togo; Ministere de la Sante et de la Protection Sociale, Angle avenue Sarakawa et avenue du 2 Fevrier, BP 336 Lomé, Togo; Service de Pediatrie, Centre Hospitalier Regional de Sokode, BP 187 Lomé, Togo; Programme National de Lutte contre le Paludisme, Quartier Administratif, BP 518 Lomé, Togo; Service des Laboratoires, Centre Hospitalier Universitaire Sylvanus Olympio, 198 rue de l’Hopital, Tokoin Hopital, BP 57 Lomé, Togo; Global Malaria Programme, World Health Organization, 20 Avenue Appia, 1211 Geneva 27, Switzerland

**Keywords:** Artemisinin combination therapy, Malaria, Treatment failure, Togo, Artemisinin, Efficacy, *k13*

## Abstract

**Background:**

Since 2005, the Togo National Malaria Control Programme has recommended two different formulations of artemisinin-based combination therapy (ACT), artesunate–amodiaquine (ASAQ) and artemether-lumefantrine (AL), for the treatment of uncomplicated malaria. Regular efficacy monitoring of these two combinations is conducted every 2 or 3 years. This paper reports the latest efficacy assessment results and the investigation of mutations in the *k13* propeller domain.

**Methods:**

The study was conducted in 2012–2013 on three sentinel sites of Togo (Lomé, Sokodé and Niamtougou). Children aged 6–59 months, who were symptomatically infected with *Plasmodium falciparum*, were treated with either AL (Coartem^®^, Novartis Pharma, Switzerland) or ASAQ (Co-Arsucam^®^, Sanofi Aventis, France). The WHO standard protocol for anti-malarial treatment evaluation was used. The primary end-point was 28-day adequate clinical and parasitological response (ACPR), corrected to exclude reinfection using polymerase-chain reaction (PCR) genotyping.

**Results:**

A total of 523 children were included in the study. PCR-corrected ACPR was 96.3–100 % for ASAQ and 97–100 % for AL across the three study sites. Adverse events were negligible: 0–4.8 % across all sites, for both artemisinin-based combinations. Upon investigation of mutations in the *k13* propeller domain, only 9 (1.8 %) mutations were reported, three in each site. All mutant parasites were cleared before day 3. All day 3 positive patients were infected with *k13* wild type parasites.

**Conclusions:**

The efficacy of AL and ASAQ remains high in Togo, and both drugs are well tolerated. ASAQ and AL would be recommended for the treatment of uncomplicated malaria in Togo.

## Background

Despite several decades of malaria control efforts, malaria-related mortality and morbidity remains a public health problem in sub-Saharan countries [1, 2]. In Togo, *Plasmodium falciparum* malaria transmission is stable, but with seasonal outbreaks which are equatorial-type in the south and tropical-type in the north of the country. Since 2005, the National Malaria Control Programme (NMCP) has recommended two different forms of artemisinin-based combination therapy (ACT) for the treatment of uncomplicated malaria: artemether–lumefantrine (AL) and artesunate–amodiaquine (ASAQ) [3]. Severe malaria is treated with injectable artesunate or quinine. To verify the continued efficacy of these two artemisinin-based combinations in Togo, efficacy monitoring is conducted every 2 or 3 years [4]. On previous evaluations conducted in 2005, 2007 and 2009, adequate clinical and parasitological response (ACPR), corrected for reinfection by polymerase chain reaction (PCR) genotyping was about 95 % on all sentinel sites [3].

At a global level, increases in the availability and use of ACT, together with the increased use of insecticide-treated bed nets, have substantially reduced malaria burden. Between 2000 and 2015, malaria incidence rates decreased by 37 % globally, and by 42 % in Africa. During this same period, malaria mortality rates fell by 60 % globally and by 66 % in the African Region [2]. This progress in malaria control is threatened by the rapid development and spread of artemisinin resistance [5]. Artemisinin resistance is characterized by slow parasite clearance [6, 7] due to the reduced susceptibility of ring-stage parasites [8–12]. Point mutations in the propeller region of a *P. falciparum* kelch (*k13*) protein have recently been linked with artemisinin resistance [13].

This article reports the results of the latest ACT efficacy evaluation carried out in 2012–2013 on three sentinel sites, and results from the investigation of mutations in the *k13* propeller domain.

## Methods

**Fig. 1:**
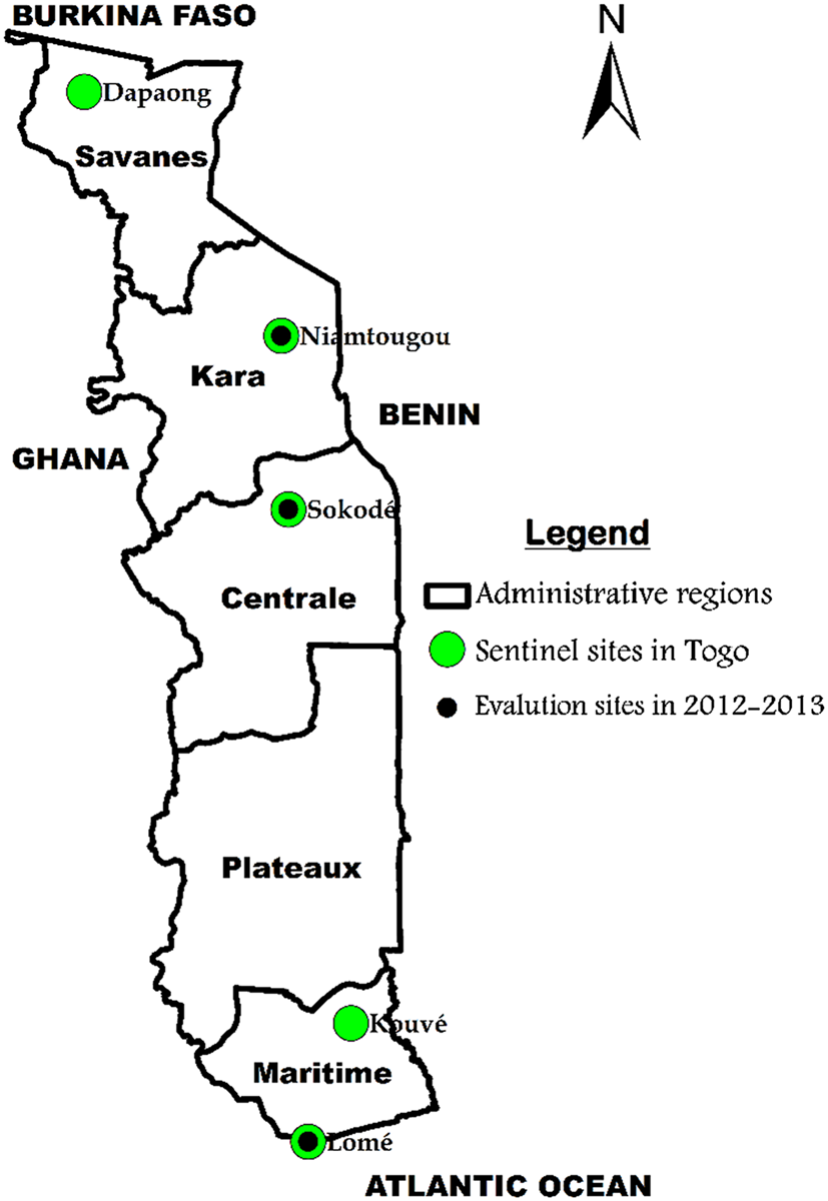
Sentinel sites for surveillance of the efficacy of artemisinin-based combination therapy in Togo

This evaluation was conducted in Togo on three of the five sentinel sites designated for the surveillance of anti-malarial treatments (Fig. 1): (1) Cacaveli Medical Centre and the District No. 2 Hospital in Lomé (the capital city); (2) the Regional Hospital and the Polyclinic in Sokodé (350 km north of Lomé) and (3) Doufelgou District Hospital in Niamtougou (425 km north of Lomé). The study took place from October to December 2012 in Niamtougou and Sokodé, to coincide with the high malaria transmission season in the north, and from June to August 2013 in Lomé.

## Study design

This was a prospective study of the clinical and parasitological efficacy of ASAQ and AL for the treatment of uncomplicated *P. falciparum* malaria, conducted according to the World Health Organization (WHO) standard protocol [14].

The sample size calculation was based on an assumed efficacy of 95 % for both, artemisinin-based combinations a 10 % significance level and a power of 95 %, with 20 % in addition, to account for patients who are likely to be either lost during follow-up, withdraw or be excluded after detection of reinfection with PCR correction [15, 16].

## Patients and treatments

Eligible patients were children aged between six and 59 months, presenting to the participating health facilities with fever or a history of fever (oral ≥37.5 °C, rectal ≥38 °C) in the last 24 h, who had microscopically-confirmed *P. falciparum* mono-infection (parasite density 2000–250,000 asexual parasites/mm^3^). Children with severe malaria symptoms according to the WHO case definition, and symptoms of severe malnutrition and chronic diseases, or with mixed *Plasmodium* infection were excluded.

Patients were randomly assigned to receive AL or ASAQ, with all doses administered under medical supervision. AL (Coartem^®^, Novartis Pharma, Switzerland) was administered as 20 mg artemether/120 mg lumefantrine fixed-dose combination tablets twice daily during 3 days according to body weight: one tablet for 5–14 kg and two tablets for 15–24 kg. ASAQ (Co-Arsucam^®^, Sanofi Aventis, France), artesunate (25 or 50 mg) and amodiaquine (67.5 or 135 mg), were administered once daily during 3 days according to body weight: 25 mg artesunate/67.5 mg amodiaquine tablets for 5–8 kg and 50 mg artesunate/135 mg amodiaquine tablets for 9–17 kg. As antipyretic, 60 mg/kg of Paracetamol per day during the first 3 days (day 0 to day 2) was given systematically to every child included in the study.

Children enrolled in the cohort received drug treatment on days 0, 1 and 2, with follow up on days 3, 7, 14, 21 and 28. Adverse events were screened and recorded at each visit. Clinical examination, including fever assessment, was performed on days 1, 2, 3, 7, 14, 21 and 28. Capillary blood was taken for parasite counts and assessment of haemoglobin levels were performed at screening (day 0) and at days 2, 3, 7, 14, 21 and 28. Haemoglobin level was measured using a point-of-care testing: the HemoCue^®^ Hb 301 System (HemoCue AB, Ängelholm, Sweden). Asexual parasitaemia was determined from Giemsa-stained thick blood smears against the number of parasites per 200 white blood cells on day 0 and 1000 at follow-up assessments, based on a putative count of 6000 white blood cells per microlitre of blood. Gametocytes were similarly enumerated [14].

Dried blood spots were obtained for PCR at enrolment (day 0) and on follow-up days 1, 2, 3, 7, 14, 21 and 28. In the case of parasitaemia detected after day 7, to distinguish between recrudescence and reinfection, PCR genotyping was performed on paired dried blood spots to determine polymorphisms in merozoite surface protein-1 (*msp*-*1*), merozoite surface protein-2 (*msp*-*2*), and glutamate-rich protein (*glurp*) polymorphism, as per WHO methods [15].

To assess the treatment effect on parasites mutations that modulate treatment response, mutations in *k13* propeller gene—the molecular marker associated with decrease in *P. falciparum* artemisinin sensitivity were investigated [5, 13]. The resistance was investigated by examining polymorphisms in the *k13* propeller domain at day 0. The method used was a nested polymerase chain reaction (PCR) protocol followed by Sanger sequencing using primers specific to *P. falciparum*. The amplicon used for sequencing covered 740 bp, which included the *k13* propeller domain [13].

## Outcomes evaluation and statistical analysis

Treatment outcomes were classified based on parasitological and clinical outcomes assessment as recommended by the WHO [14]. Therapeutic responses on day 28 were classified as either adequate clinical and parasitological response (ACPR), or treatment failure (TF); designated as early treatment failure (ETF), late clinical failure (LCF), or late parasitological failure (LPF).

The primary outcome measure was ACPR, corrected for reinfection using PCR genotyping at day 28. Patients were excluded from the analysis for the following reasons: lost to follow-up, protocol violations, treatment failure due to reinfection, or type of treatment failure could not be determined using PCR. Severe malaria cases developed during follow-up were treated with parenteral quinine according to the NCMP recommendations.

Data management and analysis were completed with Epi Info 6.0.4 adapted to the WHO’s Microsoft^®^ Excel-based applications [17] that enabled results be analysed with per-protocol and Kaplan–Meier methods.

## Results

**Fig. 2:**
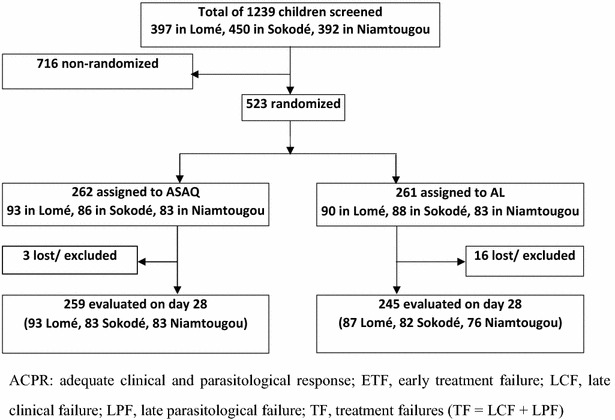
Study flow

Among 1239 children with fever screened, 523 were included in the study (Fig. 2), 50.4 % had negative blood smear, and 92 (7.4 %) were not included for the following reasons: 63 (5.1 %) for parasitaemia below 2000 parasites/mm^3^, 2 (0.2 %) for parasitaemia >250,000 parasites/mm^3^, 3 (0.2 %) for signs of severe malaria, 11 (0.9 %) for mixed *P. falciparum* and *Plasmodium malariae* or *Plasmodium ovale* infection, 6 (0.5 %) for parents refusal to consent, and 7 (0.6 %) for children refusal to take medicines.

Mean age was 37.6 and 36.6 months, respectively, for ASAQ and AL on the Lomé site, 35.5 months for both combinations on the Sokodé site, and 37.8 and 36.9 months, respectively, for ASAQ and AL on the Niamtougou site. At enrolment, the mean temperature was between 38.5 and 39.2 °C and geometric mean parasitaemia between 23,906 and 38,720/mm^3^ for all sites and treatment groups. Across all sites and treatment groups, the sex-ratio was around 1:1 (Table 1).

**Table 1:** General characteristics of study participants for treatment with ASAQ and AL by study site in Togo, 2012–2013

Drug	Site	N	Male/female	Mean age, months (SD)	Mean temperature, °C (SD)	Geometric mean parasitaemia, µL^−1^ (95 % CI)
ASAQ	Lomé	93	44/49	37.6 (16.2)	39.1 (0.9)	23,906 (2187–198,250)
ASAQ	Sokodé	86	39/47	35.6 (15.1)	38.8 (0.9)	38,720 (2026–240,000)
ASAQ	Niamtougou	83	48/35	37.8 (15.4)	38.6 (1.0)	34,242 (2226–235,000)
AL	Lomé	90	49/41	36.6 (16.8)	39.1 (0.9)	29,986 (2076–215,786)
AL	Sokodé	88	51/37	35.5 (16.5)	38.8 (0.9)	31,824 (2060–230,211)
AL	Niamtougou	83	43/40	36.9 (15.3)	38.5 (1.0)	29,685 (2172–224,250)

Fever clearance was observed by day 1 for the majority of participants; the mean temperature decreased from 38.6 °C on day 0 to 37 °C on day 1 for both combinations across all sites. On day 1, the proportion of children with mean temperatures below 37 °C were 79 and 72 % in Sokodé, 77 and 65 % in Niamtougou and 74 and 56 % Lomé, respectively, for ASAQ and AL.

**Fig. 3:**
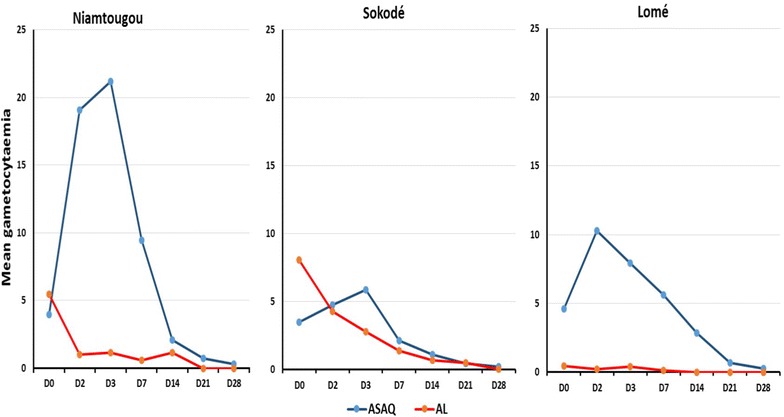
Gametocyte clearance by ACT and study site in Togo, 2012–2013

**Fig. 4:**
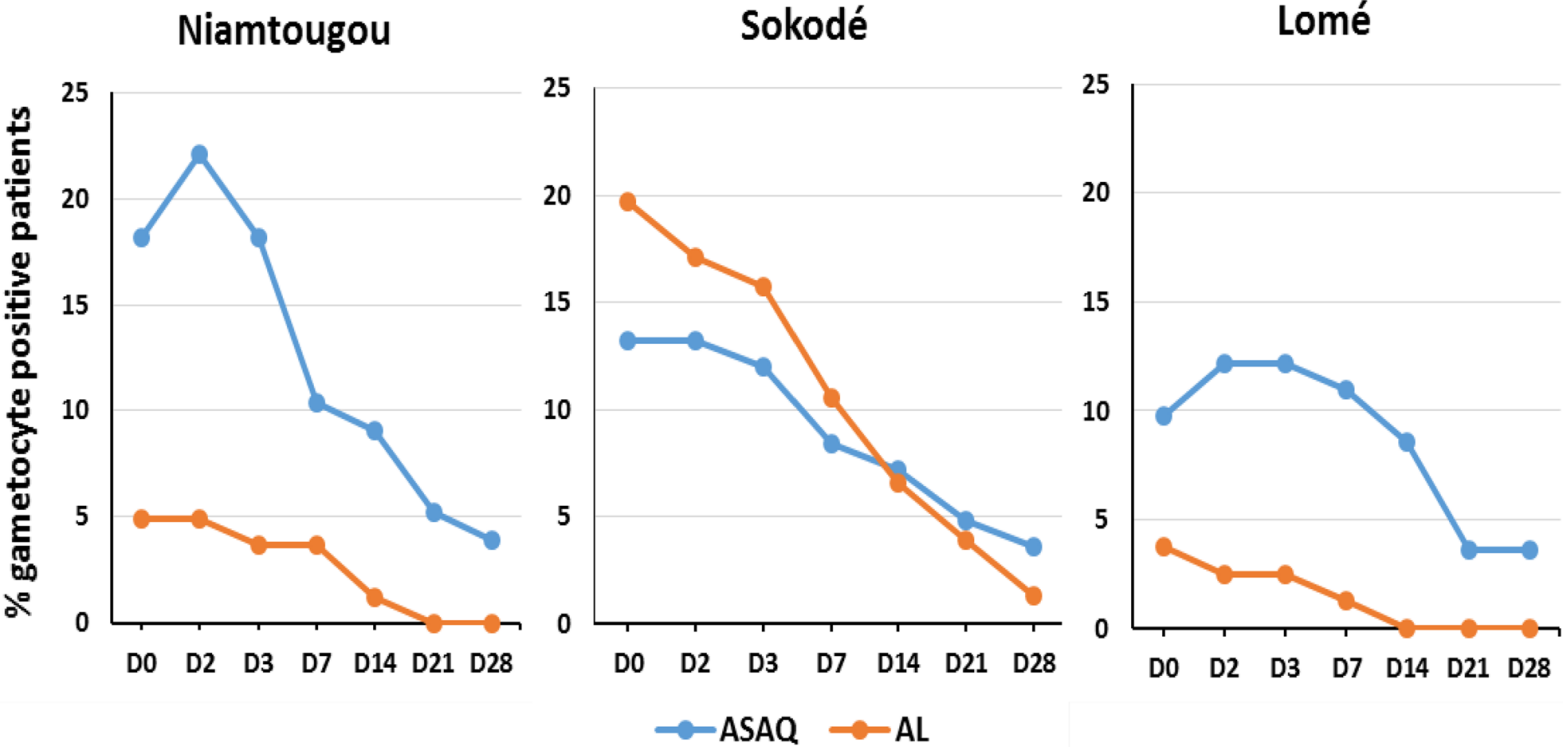
Proportion of patients that were gametocyte positive from day 0 to day 28, by ACT and study site in Togo, 2012–2013

**Fig. 5:**
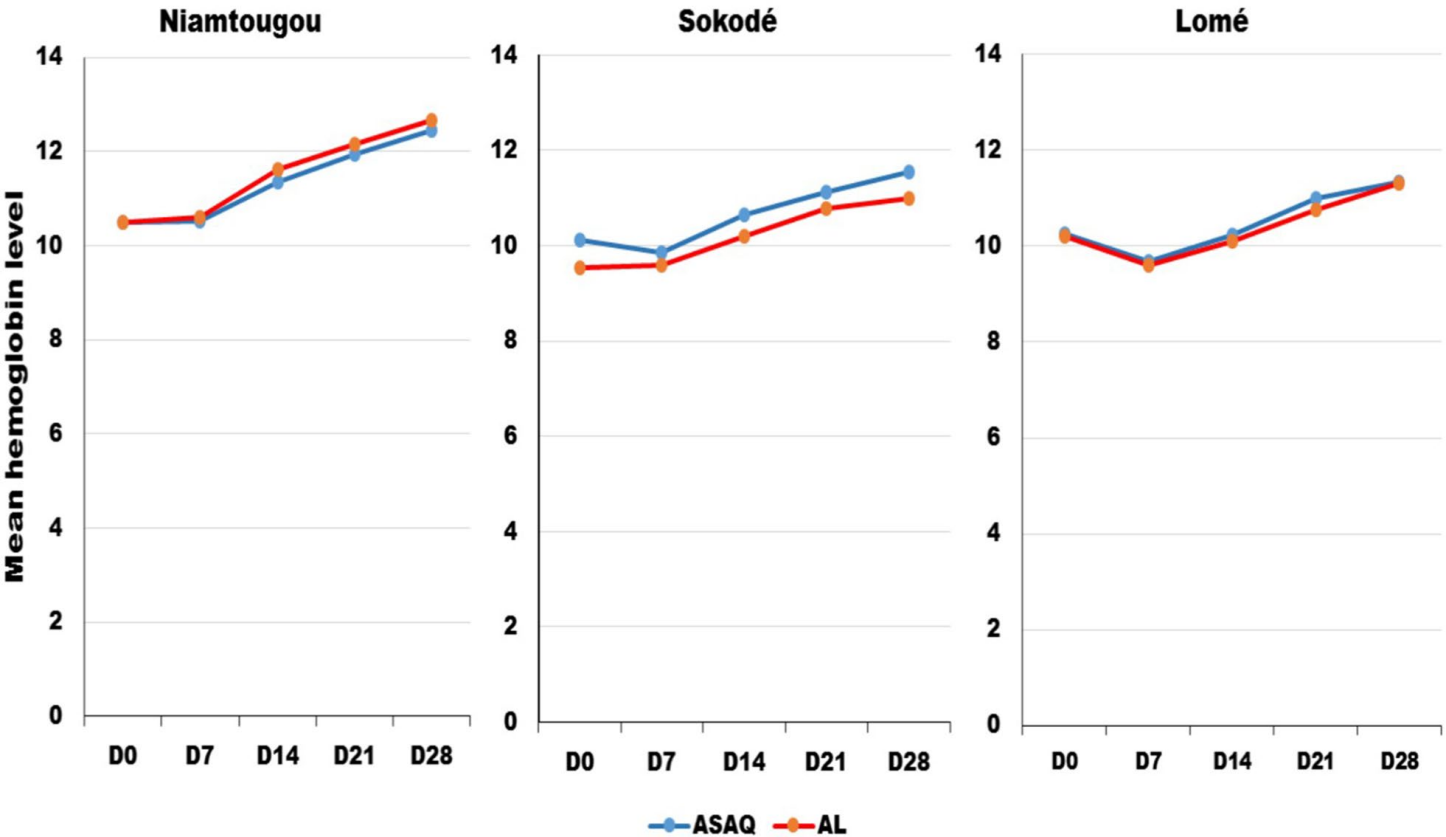
Change in haemoglobin level at enrolment and during follow-up by ACT and study site in Togo, 2012–2013

On day 1, parasite clearance was observed in 90 % of children in Sokodé, 77 % in Niamtougou and 92 % in Lomé for ASAQ and 92 % of in Sokodé, 86 % in Niamtougou and 90 % in Lomé for AL. Parasite clearance was achieved by day 3 in all patients in Sokodé and Lomé. However, at day 3, parasite clearance was achieved in Niamtougou for 94 % of patients receiving ASAQ and 96 % of those receiving AL. Gametocyte clearance was similar across the three study sites. For the AL treatment group, gametocytes decreased from day 0 throughout the follow-up days. However, for ASAQ gametocytes increased until day 3 before starting to decrease, with clearance achieved by day 28 (Fig. 3). The proportion of patients that were gametocyte positive also decreased with a similar trend as the gametocyte clearance (Fig. 4). A consistent increase in mean haemoglobin levels of 1.5–2 g/dL by day 28 was observed for both artemisinin-based combinations across the three sites (Fig. 5).

Non-PCR-corrected ACPR decreased from the southern to the northern part of the country (Table 2). AL efficacy rates were 95.4 % in Lomé, 87.8 % in Sokodé and 85.5 % in Niamtougou. ASAQ efficacy rates decreased from 97.8 % in Lomé to 97.6 % in Sokodé and to 92.8 % in Niamtougou. PCR-corrected efficacy rates varied from 96.3 to 100 % for ASAQ and 97 to 100 % for AL. Treatment failure was slightly higher for AL as compared to ASAQ (Table 2). Niamtougou is the only site where PCR-corrected treatment failure for ASAQ was recorded. Most of the treatment failures (LCF and LPF) were observed on day 14 for AL on Sokodé and Niamtougou sites. Treatment failures for ASAQ only appeared by the day 21, while on the Lomé site, the first failures were observed on day 21 for AL and day 28 for ASAQ.

**Table 2:** Parasitological and clinical outcomes among patients treated for uncomplicated *P. falciparum* malaria with either artesunate-amodiaquine (ASAQ) or artemether-lumefantrine (AL) in 2012–2013 on three sites in Togo

Drug	Site	n	Non-PCR-corrected						PCR-corrected							
			Excl/loss	ACPR, n (%)	LCF	LPF	TF (%)	(95 % CI)	Excl/loss	ACPR, n (%)	LCF	LPF	TF (%)	(95 % CI)	Kaplan–Meier	AE (%)
ASAQ	Lomé	93	0	91 (97.8)	0	2	2 (2.2)	(0.3–7.6)	2	91 (100)	0	0	0 (0.0)	(0.0–4.0)	0.0 (0.00–0.00)	1 (1.0)
ASAQ	Sokodé	86	3	81 (97.6)	1	1	2 (2.4)	(0.3–8.4)	5	81 (100)	0	0	0 (0.0)	(0.0–4.5)	0.0 (0.00–0.00)	2 (2.3)
ASAQ	Niamtougou	83	0	77 (92.8)	0	6	6 (7.2)	(2.7–15.1)	3	77 (96.3)	0	3	3 (3.8)	(0.8–10.6)	0.03 (0.01–0.10)	1 (1.2)
ASAQ	Overall	262	3	249 (96.1)	1	9	10 (3.9)	(1.9–7.0)	10	249 (98.8)	0	3	3 (1.2)	(0.2–3.4)	1.2 (0. 4–3.6)	4 (1.5)
AL	Lomé	90	3	83 (95.4)	1	3	4 (4.6)	(1.3–11.4)	7	83 (100)	0	0	0 (0.0)	(0.0–4.3)	0.00 (0.00–0.00)	0 (0.0)
AL	Sokodé	88	6	72 (87.8)	3	7	10 (12.2)	(6.0–21.3)	14	72 (97.3)	0	2	2 (2.7)	(0.3–9.4)	0.03 (0.01–0.10)	3 (3.4)
AL	Niamtougou	83	7	65 (85.5)	1	10	11 (14.5)	(7.5–14.5)	16	65 (97.0)	0	2	2 (3.0)	(0.4–10.4)	0.03 (0.01–0.10)	4 (4.8)
AL	Overall	261	16	220 (89.8)	5	20	25 (10.2)	(4.7–6.7)	37	220 (98.2)	0	4	4 (1.8)	(0.5–4.5)	1.7 (0.6–4.4)	7 (2.7)

There were no cases of early treatment failure

*ACPR* adequate clinical and parasitological response, *LCF* late clinical failure, *LPF* late parasitological failure, *TF* treatment failures (TF = LCF + LPF), *AE* adverse events

Treatment adverse events recorded during the study were mild and of low percentage: among the 262 children treated with ASAQ and 261 treated with AL, only 4 (1.5 %) and 7 (2.7 %) cases of mild adverse events, respectively in the ASAQ and AL cohorts (Table 3).

**Table 3:** Adverse events recorded following children treatment with ASAQ (n = 262) and AL (n = 261) in Togo, 2012–2013

	ASAQ		AL	
	n	(%)	n	(%)
Number of children	262	100	261	100
Adverse events				
Diarrhoea	1	(0.38)	2	(0.77)
Abscess	0	(0.00)	1	(0.38)
Asthenia	1	(0.38)	1	(0.38)
Anorexia	1	(0.38)	0	(0.00)
Haemoglobinuria	0	(0.00)	1	(0.38)
Itching	1	(0.38)	1	(0.38)
Eyelid oedema	0	(0.00)	1	(0.38)
Total	4	(1.53)	7	(2.68)

Among the 523 patients, 500 isolates were successfully sequenced for the *k13* propeller domain. Overall, 491 (98.2 %) were wild type or presented with synonymous mutations. Only 9 (1.8 %) mutations were reported, three in each site: S522M, A578S and C532S in Lomé; S522C, A578S in Sodoké; and S522C and A578S (n = 2) in Niamtougou. All the patients carrying mutant parasites cleared their parasites before day 3. In addition, all day 3 positive patients were infected with *k13* wild type parasites.

## Discussion

Following the WHO guidelines, the NMCP of Togo is recommending AL and ASAQ for the treatment of uncomplicated malaria, since 2005. A monitoring of these two artemisinin-based combinations is conducted every 2 or 3 years to provide timely information on trends of their efficacy and safety to enhance evidence-based decision making by the NMCP [3, 4].

The results showed that the two combinations induced fever clearance as quickly as day 1 following the treatment for more than 50 % of participants across all study sites. Complete parasite clearance was observed by day 3 for all participants on two sentinel sites (Sokodé and Lomé). Moreover, the gametocytocidal effect was observed by the rapid reduction in gametocytes, as early as day 1 for AL and from day 3 for ASAQ. With ASAQ, the initial increase in the mean gametocytaemia before its rapid decrease from day 3 suggests the appearance of new gametocytes following initiation of the treatment. Nevertheless, complete clearance was achieved by day 28 for both combinations. Parasites and gametocytes clearances are consistent with the significant improvement of haemoglobin levels following treatment.

Beside fever, parasite and gametocytes clearance, and improvement of haemoglobin level, efficacy results obtained in our study were also very satisfying. The PCR-corrected therapeutic efficacy of the two forms of artemisinin-based combinations recommended by the NMCP for uncomplicated malaria cases management in Togo remains above the 95 % efficacy threshold recommended by the WHO [14]. In addition, the two drugs were both well tolerated as less than 5 % of children displayed some mild adverse events consisting of diarrhoea, asthenia, anorexia, haemoglobinuria, itching, and eyelid oedema.

Studies conducted in the same settings in Togo between 2005 and 2009 showed the same significant improvement of haemoglobin levels following treatment. Moreover, the gametocytocidal effect of ACT was observed by the initial, rapid reduction in gametocytes. They also showed a high therapeutic efficacy: adequate clinical and parasitological response (ACPR), corrected for reinfection by PCR genotyping was about 95 % on all sentinel sites. In fact, the PCR-corrected 28-day cure rates using the per-protocol analysis were between 94–100 % for ASAQ and 96–100 % for AL [3].

The efficacy assessment of ACT has also shown a high efficacy level of the combinations in other West Africa countries. In Ghana, a bordering country of Togo, Abuaku et al. [18] in a study conducted in 2011, found satisfying fever, gametocytes and parasites clearance, but in two out of three study sites, and no significant improvement in haemoglobin level was observed. PCR-corrected cure rates on day 28 were 95.4 %. In contrast, another study conducted by Abuaku et al. [19] in 2014, demonstrated a satisfactory clearance of fever, gametocytes, and parasites, but significant increase in haemoglobin level. The efficacy rates using the 28 day per-protocol analysis were above 95 %: overall PCR-corrected cure rates of 100 % for ASAQ and 97.6 % for AL.

In Benin, another bordering country, a 42-day therapeutic efficacy study of AL conducted by Ogouyèmi-Hounto et al. [20] in 2014 in the northwest of the country, showed that fever was significantly cleared by day 1, about 90 % of parasites were cleared on day 1 and haemoglobin level slightly increased with parasitic clearance. The PCR-corrected cure rate was 100 %.

Results of this study are close to those of other similar studies conducted in the sub-Saharan African region during the last 5 years. These studies also showed high efficacy of ACT adopted in the area to treat uncomplicated malaria. Mekonnen et al. [21] in a study conducted in 2011 in Southwestern Ethiopia, found 97.8 % PCR-corrected cure rate for AL, with 2.2 % of 28-day gametocyte carriage rate. Oguche et al. [22] noted in their study conducted in 2010 in Nigeria, 98.3 and 96.9 % PCR-corrected cure rates for ASAQ and AL respectively, in addition the significant fever and gametocytes clearance, and improvement in haematocrit levels. However, much higher proportions of adverse events were reported as compared to those found in the present study: 37.50 % of adverse events in the AL and 46.22 % in the ASAQ treatment groups. Ojurongbe et al. [23], in a randomized comparative study at the Wesley Guild Hospital, Ilesa (Nigeria), found 100 % PCR-corrected cure rates for both ASAQ and AL. Complete fever and parasites clearance were observed by day 2 and day 3, respectively. But unlike this study, much higher proportions of adverse events were also reported: 14.8 and 21.8 % of adverse events in the AL and ASAQ treatment groups, respectively. Upon a review of nine therapeutic efficacy trials conducted in Tanzania, Shayo et al. [24] found that the efficacy of both AL and ASAQ in Tanzania was high with PCR-corrected cure rates of 91–100 and 88–93.8 %, respectively.

Although such high levels of ACT efficacy are found in sub-Saharan Africa more than 10 years after its adoption in the region, there is a concern of emergence of artemisinin resistance in Africa. So far, artemisinin resistance is confined and has been detected in five countries in the Greater Mekong Subregion (Cambodia, Lao People’s Democratic Republic, Myanmar, Thailand and Vietnam) [5, 6]. In this region, C580Y, R539T, Y493H I543T and F446I mutations are most frequently reported [25]. This study did not report any mutations associated with artemisinin resistance [26]. The A578S mutation that has been frequently reported in different countries in Africa, but in vitro studies confirmed that this specific mutation is not related to artemisinin resistance. Other mutations were rarely reported and do not seem to expand clonally in the region. The data presented here do not suggest emergence of artemisinin resistance in Togo despite increase day-3 positivity rate in Niamtougou.

## Conclusions

Both artesunate–amodiaquine (ASAQ) and artemether–lumefantrine (AL) have been shown to be safe and highly effective in the treatment of uncomplicated *P. falciparum* malaria for children in Togo. No evidence of the emergence of artemisinin resistance in Togo was found upon investigation of mutations in the *k13* propeller domain. The use of these two combinations (ASAQ and AL) adopted by health authorities, could be continued for the treatment of uncomplicated malaria in Togo.

## Abbreviations

ACPR: adequate clinical and parasitological response
ACT: artemisinin-based combination therapy
TF: treatment failure
ETF: early treatment failure
LCF: late clinical failure
LPF: late parasitological failure
AE: adverse events
AL: artemether–lumefantrine
ASAQ: artesunate–amodiaquine
*glurp*: glutamate-rich protein
*k13*: *P. falciparum* kelch (*k13*) protein
*msp*-*1*: merozoite surface protein-l
*msp*-*2*: merozoite surface protein-2
NMCP: National Malaria Control Program
PCR: polymerase chain reaction
WHO: World Health Organization
